# Growth factors‐based platelet lysate rejuvenates skin against ageing through NF‐κB signalling pathway: In vitro and in vivo mechanistic and clinical studies

**DOI:** 10.1111/cpr.13212

**Published:** 2022-03-11

**Authors:** Ting Li, Haishan Lu, Li Zhou, Ming Jia, Lei Zhang, Huiling Wu, Letian Shan

**Affiliations:** ^1^ The First Affiliated Hospital, College of Medicine Zhejiang University Hangzhou China; ^2^ Department of Plastic and Aesthetic Center The First Affiliated Hospital of Zhejiang University Hangzhou China; ^3^ Department of Dermatology PLA 903 Hospital Hangzhou China; ^4^ The First Affiliated Hospital Zhejiang Chinese Medical University Hangzhou China; ^5^ Affiliated Hangzhou First People's Hospital Zhejiang University School of Medicine Hangzhou China; ^6^ Cell Resource Bank and Integrated Cell Preparation Center of Xiaoshan District Hangzhou Regional Cell Preparation Center (Shangyu Biotechnology Co., Ltd) Hangzhou China

## Abstract

**Introduction:**

Platelets benefit tissue regeneration by secreting growth factors, and platelet products, for example, platelet lysate (PL), have been clinically applied for tissue rejuvenation. To determine the anti‐ageing efficacy and mechanism of human PL (hPL) on skin, this study conducted clinical retrospective analysis, nude mice‐based in vivo study and human dermal fibroblasts (HDFs)‐based in vitro study.

**Methods:**

Flow cytometry was employed for quality control of hPL, and ELISA was used for quantification of growth factors (EGF, IGF‐1, PDGF and TGF‐β) in hPL. After d‐galactose modelling, skin texture grading, histopathological observation, immunofluorescence analysis and oxidative stress assays were conducted on nude mice, while SA‐β‐gal staining, CCK‐8 and wound healing assays were conducted on HDFs. qPCR and western blot were conducted to clarify hPL's mechanism.

**Results:**

The clinical retrospective data showed that hPL obviously rejuvenated human skin appearances without adverse events. The animal data showed that hPL exerted rejuvenative effects on skin, and the cellular data showed that hPL significantly promoted the proliferation and migration of HDFs and suppressed senescence‐associated secretory protein secretion and senescence state of senescent HDFs by suppressing NF‐κB pathway. The NF‐κB‐dependent mechanism was verified positively by using P65 siRNA and negatively by using prostratin. Furthermore, EGF, IGF‐1, PDGF and TGF‐β were found as the main ingredients in hPL, which contributed to the efficacy and mechanism of hPL.

**Conclusion:**

This study provided novel knowledge of hPL, making it ideal for skin rejuvenation.

## Graphical abstract

Human platelet lysate (hPL) exerted rejuvenative and regenerative efficacy on skin ageing of nude mice and senescent HDFs through NF‐κB signalling pathway. A retrospective study further verified hPL's efficacy and safety in clinic.

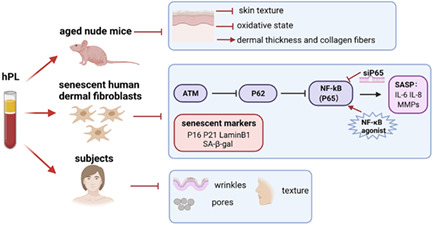

## 1 INTRODUCTION

Skin ageing is the most intuitive consequence of ageing, affecting epidermis, dermis and subcutaneous layer.^1^ Dermis layer, containing collagen fibres, elastic fibres and other extracellular matrix (ECM), suffers the most changes of ageing in skin.^2^ Human dermal fibroblasts (HDFs) are the primary cell types in dermis layer and are responsible for synthesis of collagen and elastic fibres.^3^ With ageing, the quantity and proliferation rates of HDFs are reduced. Once aged, HDFs lose their capacities to produce collagen and release senescence‐associated secretory proteins (SASP) and matrix‐degrading metalloproteinases (MMPs), resulting in degradation of ECM.^4^, ^5^ Phenotypically, skin wrinkles are increased, and meanwhile, skin elasticity and mechanical resistance are decreased.^6^ Skin ageing contains extrinsic ageing and intrinsic ageing, both of which involve inflammation, DNA damage and oxidative stress.^7^, ^8^, ^9^ Extrinsic ageing is mainly caused by environmental factors, such as UV radiation, while intrinsic ageing is closely associated with cellular senescence.^10^ Inflammation and DNA damage are the main causes of cellular senescence in the intrinsic ageing, which produce SASP (e.g., IL‐6 and IL‐8) with activation of NF‐κB signalling pathway.^11^ Moreover, reactive oxygen species in oxidative stress causes inflammatory response and MMPs overexpression, resulting in acceleration of ageing process via NF‐κB signalling pathway.^12^, ^13^ Therefore, NF‐κB signalling pathway plays predominant role in the pathogenesis of cellular senescence and SASP release of intrinsic skin ageing, acting as an important target for anti‐ageing treatment.

Many therapeutics and techniques have been explored to prevent or reverse skin ageing for several decades. Antioxidants, such as resveratrol and epigallocatechin gallate (EGCG), have been applied to scavenge ROS and suppress inflammation of aged skin.^14^, ^15^ Nonetheless, resveratrol is easily metabolized and needs to be packaged by vehicle for use, making it difficult for delivery in clinical applications.^10^ The use of EGCG lacks standard of dose range, resulting in uncertain effectiveness in clinic.^12^, ^15^ Retinoids, such as retinol and tretinoin, have been applied to treat skin ageing by regulating MMPs activity and promoting collagen production to improve skin textures.^16^, ^17^ However, retinoids have risks of inducing dermatitis and erythema in clinic, since their use with inappropriate doses may induce skin irritation.^18^ Injective techniques by using botulinum toxin are extensively employed for treating dynamic wrinkles of aged skin.^19^, ^20^ Nevertheless, this chemical may cause immediate vision loss and non‐hypersensitivity reactions.^21^, ^22^ Botulinum toxin is gradually metabolized over time and finally disappears, the effect of which cannot last for a long time.^23^ Recently, cell therapy‐based techniques have attracted increasing attention in the field of anti‐ageing. Injection of platelet‐rich plasma (PRP) is the most popular one that can improve facial skin appearance (texture and lines), increase skin thickness and enhance collagen content.^24^, ^25^ However, this technique lacks standard of preparation, contains cellular residues and cannot be stored, resulting in less than 50% global improvements rate and unsatisfactory efficacy in clinic.^25^, ^26^ Therefore, it is necessarily needed to develop new techniques with certain effectiveness for skin ageing treatment.

Platelet lysate (PL) is a growth factor‐released product of platelets and also the next generation of PRP, containing insulin‐like growth factor (IGF‐1), platelet‐derived growth factor (PDGF), epidermal growth factor (EGF), transforming growth factor‐β (TGF‐β), and so forth, by freeze–thawing preparation from platelet concentrates.^27^ PL is cell free with advantages over PRP, including: (1) the avoidance of heterologous material uses for PL preparation; (2) the availability of long‐term storage of PL in low temperature for consecutive applications and (3) stable release of high concentrations of growth factors that exerts better efficacy and can easily be quality controlled for standardization.^28^, ^29^ Previous reports showed that PL exerted regenerative effects on various tissues and cells, such as skin, cartilage, tendon cells and nerve cells.^30^, ^31^, ^32^ Recently, our studies demonstrated that PL exerted protective effects on chondrocytes and cartilage against osteoarthritis through regulation of NF‐κB signalling pathway, and that PL benefited umbilical cord‐derived MSCs (huc‐MSCs) by promoting cell proliferation, cell cycle progression and cell migration through activation of AMPK/mTOR signalling pathway‐mediated autophagy.^28^ Moreover, we found that the growth factors (PDGF, IGF‐1, TGF‐β and EGF) contributed to the regenerative effects of PL in varying degrees.^28^ Of these, PDGF and EGF are capable of promoting collagen deposition and tissue formation in the defect region of skin, resulting in re‐epithelialization and skin regeneration.^32^, ^33^, ^34^, ^35^ Therefore, PL possesses pro‐regenerative effect on skin, which has great potential for skin ageing treatment. To date, there is little study regarding the anti‐ageing mechanism of PL.

To determine the anti‐ageing efficacy and explore the underlying mechanism of human PL (hPL) on skin, we retrospectively analysed subjects treated with hPL and then established an aged skin model of nude mice and HDFs by using d‐galactose (D‐gal) for efficacy and mechanism study. D‐gal‐induced ageing model has been widely used to mimic intrinsic or natural ageing of human beings for anti‐ageing studies, which causes oxidative stress and cell senescence by triggering inflammatory processes on skin.^36^, ^37^, ^38^, ^39^ In this study, the efficacy and mechanism of hPL on skin ageing were determined for the first time, providing a promising therapeutic strategy for anti‐ageing treatment.

## 2 MATERIALS AND METHODS

### 2.1 Reagents and materials

DMEM/HIGH GLUCOSE was purchased from Gibco BRL (NY, USA). Trypsin (0.25%) was purchased from Thermo Fisher Scientific (MA, USA). Foetal bovine serum (FBS) was purchased from CellMax (Beijing, China). Radioimmunoprecipitation assay (RIPA) buffer, proteinase inhibitor cocktail and cell counting kit‐8 (CCK‐8) kit were purchased from Bimake (TX, USA). Cell culture plates were purchased from Eppendorf (Hamburg, Germany). TRIzol reagent and DNase I kit were obtained from TaKaRa Biotechnology Co., Ltd. (Dalian, China). All‐in‐One cDNA Synthesis SuperMix kit was purchased from Biotool (TX, USA). The 2× SYBR Green qPCR Master Mix (low ROX) kit was obtained from Bimake (TX, USA). Nitrocellulose membrane was bought from Sartorius Stedim Biotech (Göttingen, Germany). The antibodies were purchased from Cell Signaling Technology Inc. (MA, USA). Western Lightning® Plus ECL was obtained from Perkin Elmer, Inc. (Waltham, MA, USA) and X‐ray film was purchased from Kodak (Tokyo, Japan). Senescent cells histochemical staining kit was obtained from Beyotime Biotechnology (Shanghai, China). SiRNA of P65 and non‐targeting control siRNA were purchased from RiboBio Co., Ltd. (Guangzhou, China). Lipofectamine RNAiMAX Transfection Reagent was obtained from Thermo Fisher Scientific (Waltham, USA). Enzyme‐linked immunosorbent assay kits were bought from Lianke Biotech Co., Ltd. (Hangzhou, China). Prostratin was purchased from Abcam (ab120880). Bicinchoninic acid (BCA), malondialdehyde (MDA) detection kit and total superoxide dismutase (SOD) detection kit were provided by Nanjing Jiancheng Biological Engineering Institute (Nanjing, China). The recombinant products of IGF‐1, TGF‐β, PDGF and EGF were purchased from Peprotech (London, UK). The ELISA kits were purchased from Multi Sciences (Lianke) Biotech Co., Ltd. (Hangzhou, China).

### 2.2 Human PL preparation and quality control

hPL was obtained using a two‐step procedure: (1) extraction and purification of platelet concentrates and (2) freeze–thawing lysis to produce hPL, as described by our previous studies.^28^, ^29^ The methodology was innovative and licensed by China National Invention Patent (ZL 2014 1 0508458.8). Briefly, after obtaining informed consent from healthy adult donors, whole blood was collected in tubes containing sodium citrate anticoagulant (3.2%, w/v; blood:citrate = 9:1). To obtain purified platelet concentrates (purity >99%), each 50 ml of whole blood was centrifuged at 210×*g* for 10 min, and yellow plasma with buffy coat was collected in a new tube and centrifuged at 210×*g* for 5 min. Residual erythrocytes were discarded, the supernatant plasma and platelet pellet were collected as platelet concentrates. All blood specimens used in this study were approved by Ethical Committee of the Zhejiang Chinese Medical University.

To determine the purity of platelet in our product before lysis, the human platelet surface marker (CD41a) was analysed as previously described.^28^, ^29^ Briefly, the sample was incubated with the antibody against CD41a, followed by incubation for 1 h. Then the sample was washed with PBS and loaded on flow cytometer (BD FACS Calibur, BD Biosciences, CA, USA) in triplicates. Fluorescent signal intensity was recorded and analysed by CellQuest software (Version 3.3, BD Biosciences, CA, USA). The platelet number was measured by Mindray BC‐3000Plus Blood Cell Analyzer (Shenzhen, China) and standardized to 1 × 10^8^ platelets/ml, and then lysed by repeating freeze–thaw (−80 to 37 °C) for three times. The residual platelet fragments were removed by centrifugation and the obtained supernatant was collected as hPL. The concentrations of IGF‐1, TGF‐β, PDGF and EGF in hPL were measured in triplicates with commercially available ELISA kits, according to each manufacturer's instructions. The absorbance was measured using a microplate reader (Bio‐Rad Laboratories, Inc., Hercules, CA, USA).

### 2.3 Retrospective analysis of clinical data

A total of 30 subjects who have been treated with hPL injection from June 2019 to December 2019 on face in the 903rd Hospital of PLA were enrolled in this study. All subjects are eligible for inclusion and signed informed consent forms. The subjects received a total of three face hPL treatments at 5 ml with a 2‐month interval between treatments. We analysed the changes in skin wrinkles, pores and texture on subjects' face by VISIA® Imaging Systems (Canfield Scientific, Inc., Fairfield, NJ, USA) before and after hPL treatments. VISIA® is the most widely used imaging analysis device in dermatology and cosmetology, consisting of a camera system and imaging analysis software (Image‐Pro® Plus 7.0, Media Cybernetics Inc., Rockville, MD, USA). The photo documentations of skin physiological parameters were performed by VISIA®: pores are round openings for sweat ducts on the surface. The pores are represented by dark blue circles in VISIA® imaging system. The size of the circle corresponds to the size of the pore; wrinkles are folds or creases of the skin. The dark green lines represent the most noticeable wrinkles, while the light green lines represent fine wrinkles; skin texture is an analysis of skin smoothness. The crests and troughs on the skin indicate changes in the skin morphology. Yellow indicates the raised area and blue indicates the sunken area. In the end, the VISIA® imaging system will precisely generate the absolute scores based on different feature.

Subjects include the following criteria: (1) Subjects who were between 30 and 60 years old when obtaining informed consent. (s) Subjects who need skin rejuvenation, and have obvious skin wrinkles, texture and pores. The exclusive criteria includes: (1) Subjects who regularly go to plastic surgery for injection treatment during the treatment period. (2) Subjects who continuously take functional foods or quasi‐drugs that have the same or similar effects as PL. (3) Subjects suffering from atopic dermatitis and other skin diseases. (4) Subjects who showed obvious skin condition changes that were not related to hPL injection during the treatment. (5) Subjects who have severe medical problems. (6) Pregnancy or became pregnant during treatment.

### 2.4 Animal experiments

A total of thirty 6‐week‐old healthy SPF grade BALB/c nude male mice, weighing 18–22 g, were provided by Shanghai Slack Company, animal production licence number (SCXK: 2017‐0005, Shanghai, China). The mice were raised by the Animal Experiment Research Center of Zhejiang Chinese Medical University. All mice were housed in the controlled room with constant temperature (22 ± 2°C), humidity 50%–60%, light and dark alternately every 12 h, and allowed ad libitum food and water. All mice were treated in strict accordance with the China legislation on the use and care of laboratory animals. All experiments on the mice were approved by the Medical Norms and Ethics Committee of Zhejiang Chinese Medical University (Approval number SYXK:2018‐0012, Zhejiang, China).

The mice were randomly divided into five groups: normal control group (NC), skin ageing model group (Model), low hPL dose group (hPL‐L), medium hPL dose group (hPL‐M) and high PL dose group (hPL‐H). The model group and all hPL groups were treated with subcutaneous injection of D‐gal dissolved in 0.9% normal saline to the back at a dose rate of 1500 mg/kg/day (BW/day). NC group was treated with 0.9% normal saline for comparison. After D‐gal ageing modelling for 6 weeks, mice in hPL‐L, hPL‐M and hPL‐H groups were subcutaneously injected with 0.25 ml of dilution rates of 1/20 hPL, dilution rates of 1/40 hPL and dilution rates of 1/80 hPL, respectively. Meanwhile, NC group was subcutaneously injected with 0.25 ml of saline. All treatments were weekly conducted for 3 weeks, and thereby all the mice were sacrificed and the pieces of skin from the back were taken for histological and biochemical examination. Prior to the experiments, after modelling and hPL treatments, mice in different groups were photographed to record skin changes. Skin state was graded according to the appearances of wrinkles and textures^40^ (Table 1).

**TABLE 1:** Skin grades of nude mice

Grades	Skin state description
0	The primary line and the secondary line have the same depth, clearly visible and intersected. Wrinkles are thin and superficial.
1	The secondary lines are flattened, and the number is reduced. A few wrinkles are shallow coarse.
2	The primary line becomes uneven, the secondary line becomes obviously flat or deformed, and the number of intersections is reduced. Some wrinkles are coarse.
3	The texture is deteriorated, the primary lines are thick and deep, a large flat skin appears between the primary lines, and secondary lines are deformed and disappeared. Wrinkles are deeply coarse and wide.

### 2.5 SOD and MDA analysis

The skin tissue sample is weighed, added physiological saline to produce a 10% homogenate and sonicated twice every 30 s. The homogenization and sonication were performed at 4°C. After sonication, the homogenate was centrifuged at 3000 rpm for 10 min and at 12,000 rpm for 15 min. An aliquot of the supernatant was used for further experiments. BCA protein determination kit was used to determine the protein content of aliquots. SOD and MDA kit were used to detect the content of SOD and MDA in the skin of nude mice, according to each manufacturer's instructions. The SOD in the cell supernatants were estimated following the kit instructions.

### 2.6 Histopathological observation and immunofluorescence analysis

Each skin sample was fixed with formalin (10%) for 24 h at room temperature. Then each sample was embedded in paraffin and sectioned into 4 μm, followed by haematoxylin and eosin (HE) staining and Masson staining. The stained sections were observed under microscopy and statistically by ImageJ 1.47 software (Media Cybernetics, Bethesda, MD, USA). Unstained replicates of the sections were incubated overnight at 4°C with 1000 μl PBS‐diluted (1:1000) primary antibodies against mice VEGF for immunofluorescence. After PBS wash, the sections were incubated with horseradish peroxidase conducted secondary antibodies for 1 h at room temperature, followed by 2‐(4‐amidinophenyl)‐6‐indolecarbamidine dihydrochloride (DAPI) staining solution for 5 min. The skin sections were visualized under a fluorescence microscope (CarlZeiss, Göttingen, Germany). The immunoreactivity of VEGF was semi‐quantified by ImageJ 1.47 software (Media Cybernetics, Bethesda, MD, USA).

### 2.7 Cell culture and modelling

HDFs were provided by Kunming Cell Bank of Chinese Academy of Sciences (KCB200537). HDFs were cultured in high‐DMEM supplemented with 10% FBS and maintained at 37°C with 5% CO_2_. Senescent HDFs modelling was established by the treatment of D‐gal at 20 g/L concentration for 24 h.

### 2.8 Cell viability assay

The cell viability of HDFs was determined by CCK‐8 assay at 24 and 48 h. Cells were plated on 96‐well plates at a density of 4.5 × 10^3^ cells/well in 200 μl medium, followed by the treatment of D‐gal at 20 g/L concentration for ageing modelling. After 24 h, HDFs were treated with dilution rates of 1/10 to 1/200 gradient concentrations of hPL. Aliquots of each 10 μl CCK‐8 solution were added to each well and incubated at 37°C for 2 h, until the colour turned to orange. The optical density value was measured at 450 nm with a microplate reader (Bio‐Rad Laboratories, Inc., Hercules, CA, USA). Proliferative rate (%) = (EAA − treated OD/untreated OD) × 100. hPL at the dilution rate of 1/40 was used in the further experiment. Each experiment was conducted in triplicate.

### 2.9 Senescence‐associated‐beta‐galactosidase staining

HDFs were plated in six‐well plates (1 × 10^5^/well), modelled with D‐gal at 20 g/L for 24 h and followed by hPL at the dilution rate of 1/40 treatment for 24 h. The HDFs were thereby fixed with 4% paraformaldehyde and senescence‐associated‐beta‐galactosidase (SA‐β‐gal) stained using senescent cells staining kit, according to each manufacturer's instructions. Three images per each well were collected, and the SA‐β‐gal‐stained cells were counted by ImageJ 1.47 software. Each experiment was conducted in triplicate.

### 2.10 Wound healing assay

To conduct the wound healing assay, HDFs in the logarithmic growth phase were cultured in six‐well plates (1 × 10^5^/well) and artificially formed scratched area, followed by modelling with D‐gal at 20 g/L for 24 h, and then treatment of hPL with serum‐free medium. The cells were observed and imaged at three different time points (0, 24 and 48 h) under an inverted microscope (CarlZeiss, Göttingen, Germany). The scratched area was calculated with ImageJ 1.47 software. Each experiment was conducted in triplicate.

### 2.11 Real time PCR

The mRNA expression of targeted genes in HDFs cells was measured using a qPCR assay on an ABI QuantStudio™ 7 Flex Real‐Time PCR System (Applied Biosystems; Thermo Scientific, USA). Total RNA was extracted with TRIzol reagent and quality controlled by NanoDrop2000 spectrophotometer (Thermo Scientific, USA). cDNA reverse transcription was performed by using All‐in‐One cDNA Synthesis SuperMix. The PCR system was 20 μl, including 10 μl SYBR Green qPCR Master Mix (low ROX), 0.4 μl PCR forward primer, 0.4 μl PCR reverse primer, 1 μl template cDNA and 8.2 μl ddH_2_O, with the following reaction conditions: initial denaturation at 95°C for 5 min, 40 cycles of denaturation at 95°C for 3 s and annealing and extension at 60°C for 30 s. β‐Actin was used as reference gene and the 2^−Δ∆CT^ method was used to analyse the relative mRNA expressions (Table 2). Each experiment was conducted in triplicate.

**TABLE 2:** Primer sequences used for qPCR analysis

Gene	Forward primer	Reverse primer
*β‐ACTIN*	5′‐CCCGCGAGTACAACCTTCT‐3′	5′‐CGTCATCCATGGCGAACT‐3′
*IL‐6*	5′‐GCCACTGCCTTCCCTACTTCA‐3′	5′‐GACAGTGCATCATCGCTGTTCA‐3′
*IL‐8*	5′‐TGGCAGCCTTCCTGATTT‐3′	5′‐AGGTTTGGAGTATGTCTTTATGC‐3′
*MMP1*	5′‐GTGCAGACGCCAGAAGAATCT‐3′	5′‐TGTCACACGCTTTTGGGGTTT‐3′
*MMP2*	5′‐TACAGGATCATTGGCTACACACC‐3′	5′‐GGTCACATCGCTCCAGACT‐3′
*MMP3*	5′‐GAGGCATCCACACCCTAGGTT‐3′	5′‐TCAGAAATGGCTGCATCGATT‐3′
*P21*	5′‐GGCAGACCAGCATGACAGATT‐3′	5′‐GCGGATTAGGGCTTCCTCT‐3′
*COL2*	5′‐TGGACGCCATGAAGGTTTTCT‐3′	5′‐TGGGAGCCAGATTGTCATCTC‐3′
*TGF‐β1*	5′‐CAGAAATACAGCAACAATTCCTGG‐3′	5′‐TTGCAGTGTGTTATCCGTGCTGTC‐3′
*P16*	5′‐CATGGTGCGCAGGTTCTTG‐3′	5′‐CGGGATGTGAACCACGAAA‐3′

### 2.12 Western blot analysis

Total cellular proteins of HDFs were extracted with RIPA buffer containing proteinase inhibitor cocktail for 30 min on ice. The targeted protein was separated by denaturing sodium dodecyl sulphate polyacrylamide gel electrophoresis (8%–12%) and transferred onto a nitrocellulose membrane. The membrane was blocked with 5% non‐fat milk for 2 h, which was followed by overnight incubation at 4°C with the following primary antibodies against β‐actin, ATM, P65, p‐P65, P62, interleukin‐6 (IL‐6), P16, P21, LaminB1, MMP3 and MMP9. Following incubation with peroxidase‐conjugated goat anti‐rabbit/mouse IgG at 4°C for 2 h, each protein was visualized using Western Lightning® Plus ECL, detected using X‐ray film and scanned. Each experiment was conducted in triplicate.

### 2.13 Molecular and cellular verification of hPL mechanism

For transient knockdown of human P65 gene, siRNA of P65 (P65‐siRNA) and non‐targeting control siRNA were designed to transfect HDFs by using lipofectamine RNAiMAX Transfection Reagent, according to the manufacturer's instruction. Western blot and cell viability assays were conducted to verify the siRNA knockdown of P65 gene and the function of P65‐related mechanism of hPL on senescent HDFs. Each experiment was conducted in triplicate.

HDFs were plated on 10‐cm plates at a concentration of 1 × 10^6^ cells/well. Then prostratin (1 μM) was added for NF‐κB activation and incubated for 12 h. Western blot and cell viability assays were conducted to verify the changes of NF‐κB pathway following prostratin treatment. Each experiment was conducted in triplicate.

### 2.14 Data analysis

Data were analysed using IBM SPSS 26.0 Statistics and expressed as mean values ± SD. Data from different groups were compared using one‐way ANOVA followed by Fisher's least significant difference comparison. Data from clinical retrospective study were used paired‐sample *t*‐test. A *p*‐value <0.05 was considered to indicate a significant difference, and *p*‐value <0.01 considered to indicate a very significant difference.

## 3 RESULTS

### 3.1 Retrospective analysis of hPL on human skin ageing

**FIGURE 1:**
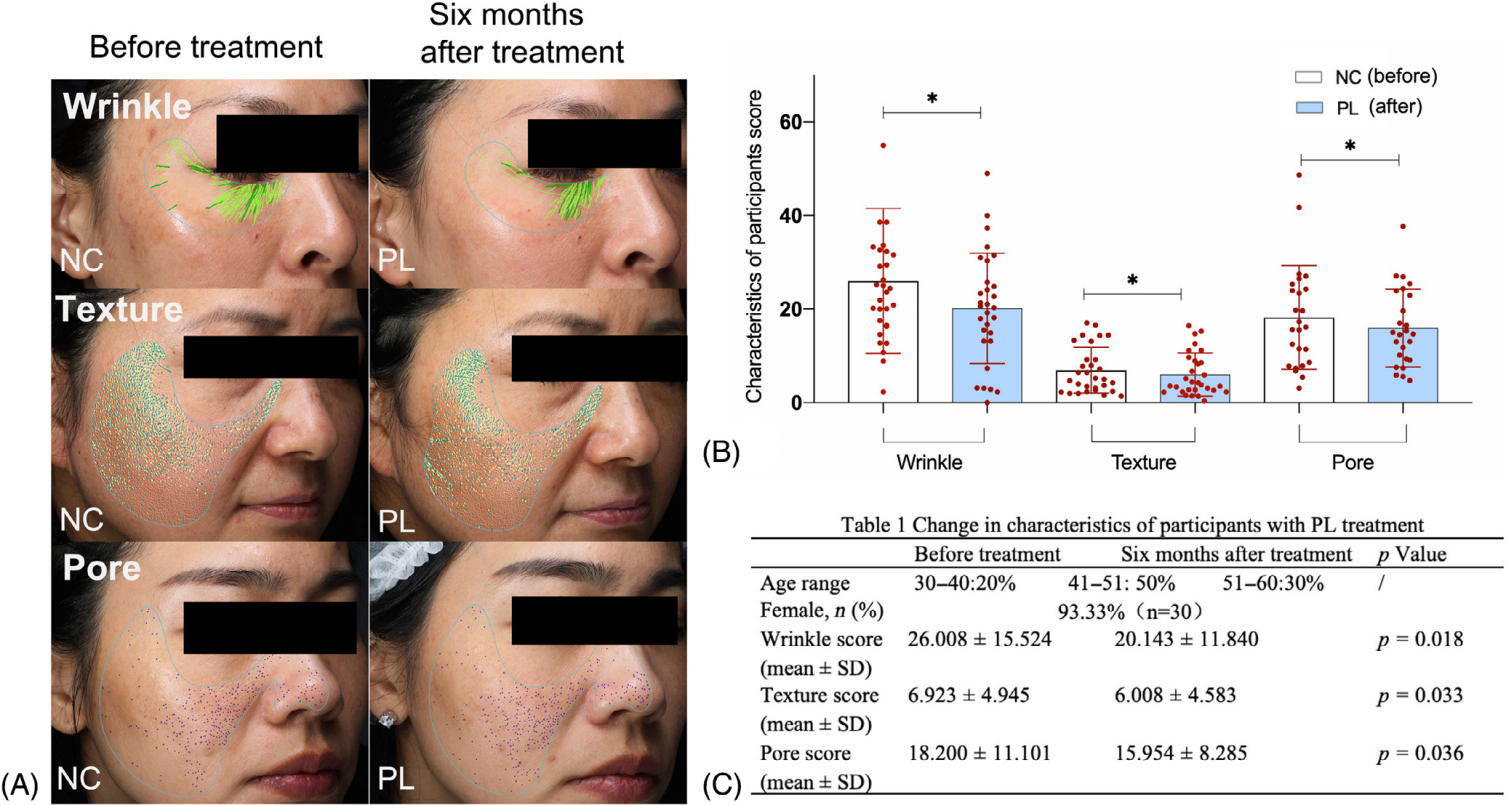
Clinical retrospective study on human platelet lysate (hPL). Wrinkle, texture and pore appearance of subjects with VISIA® imaging system before and after hPL treatment (A). Objective assessments and score of skin characteristics before and after hPL treatment (B). Data expressed as mean ± SD. *p < 0.05 vs. NC group by paired‐sample *t*‐test

Clinical efficacy of hPL was evaluated by analyses of facial wrinkles, skin texture and pores before and after hPL treatment. A total of 30 subjects who have been treated with hPL treatment were enrolled according to the above inclusive criteria. The age of subjects is between 30 and 60 years old and female contained 93.33%. The percentages of subjects in the age range of 30–40, 41–50 and 51–60 years old were 20%, 50% and 30%, respectively (Figure 1C). As shown in Figure 1A, hypodermic injection of hPL obviously decreased the deep wrinkles (represented by dark green lines) and thin wrinkles (represented by light green lines), smoothed the texture and ameliorated the sunken areas of subjects' facial skin, when compared with the parameters before hPL treatment. As shown in Figure 1B, the scores of skin characteristics of all subjects were significantly restored towards normal states by hPL (each *p* < 0.05 vs. NC). Moreover, the clinical outcomes showed that hPL did not cause any adverse event on skin or body, indicating clinical safety of this treatment.

### 3.2 In vivo effects of hPL on aged skin of nude mice

**FIGURE 2:**
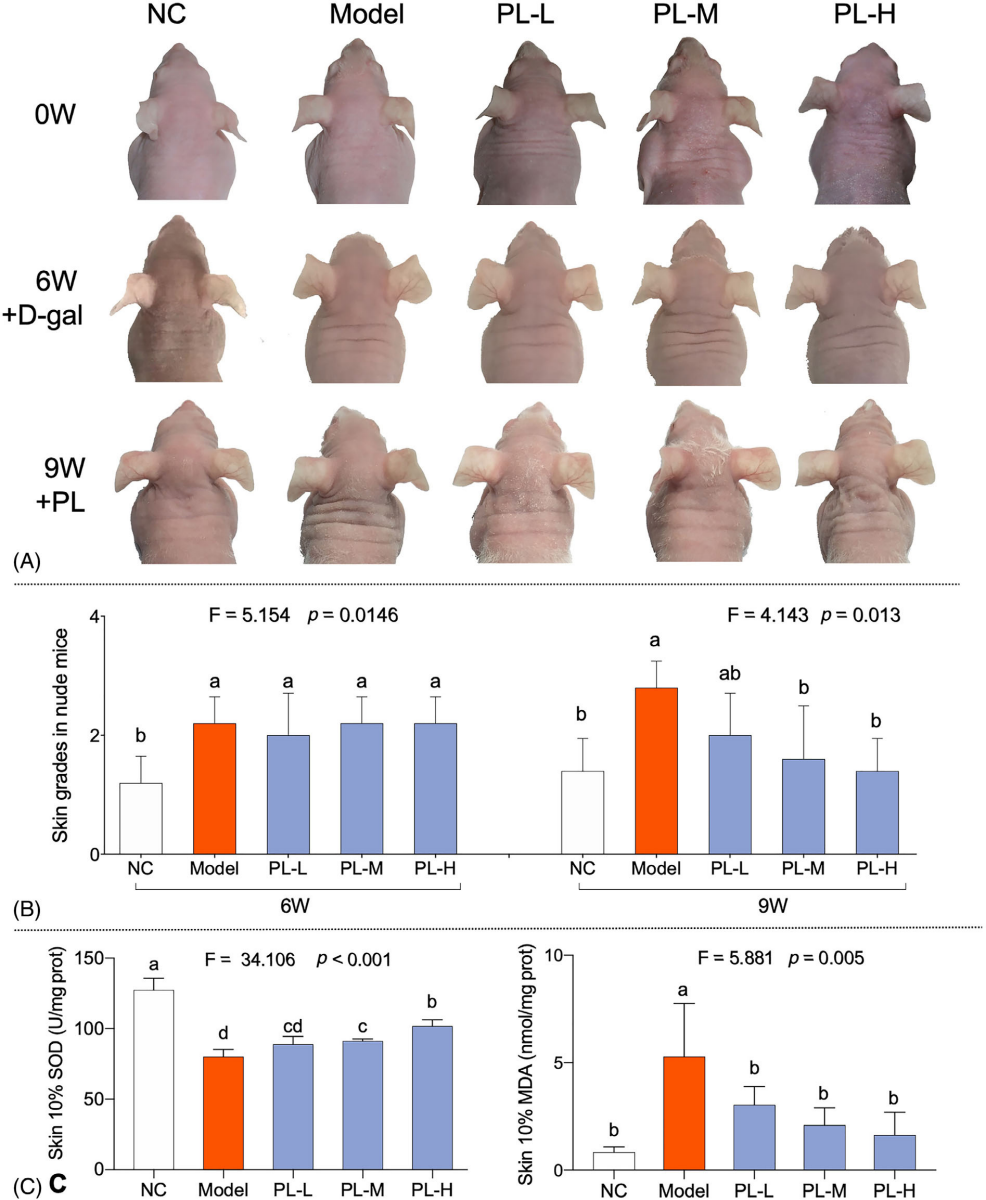
Gross skin appearance (A) and skin texture grade of mice (B); activities of superoxide dismutase (SOD) and malondialdehyde (MDA) in the skin of nude mice (C). Data expressed as mean ± SD. Data are mean values ± SD. Different symbols (a, ab, b, c, cd and d) indicate significant difference among groups (Fisher's least significant difference, *p* < 0.05), and the values decrease with the order from a to d. We repeated the experiments three times to ensure the accuracy of the experiments

To determine the in vivo effect of hPL on aged skin, D‐gal injected dorsal skin areas of nude mice were photographed and analysed by skin grading. As shown in Figure 2A,B, the skin was rough with deep and wide wrinkles in the model mice, which exhibited higher grade than that of NC group (*p* < 0.01 vs. NC). With hPL treatment for 3 weeks, the skin returned to smooth state and the wrinkles were much fainter than that of model group (*p* < 0.01 vs. model). The effect of hPL was in a dose‐dependent manner. To evaluate the antioxidant effect of hPL on aged skin, SOD and MDA activity of skin samples were measured. As shown in Figure 2C, the SOD and MDA activities of model skin samples were significantly abnormal when compared with the NC group (*p* < 0.01 vs. NC), while these abnormalities were significantly restored by hPL treatment in a dose‐dependent manner (*p* < 0.01 vs. model). The above results indicated that hPL exerted anti‐ageing effects by ameliorating textures and reversing oxidative state of aged skin.

### 3.3 Histopathological and immunofluorescence analyses of aged skin with hPL treatment

**FIGURE 3:**
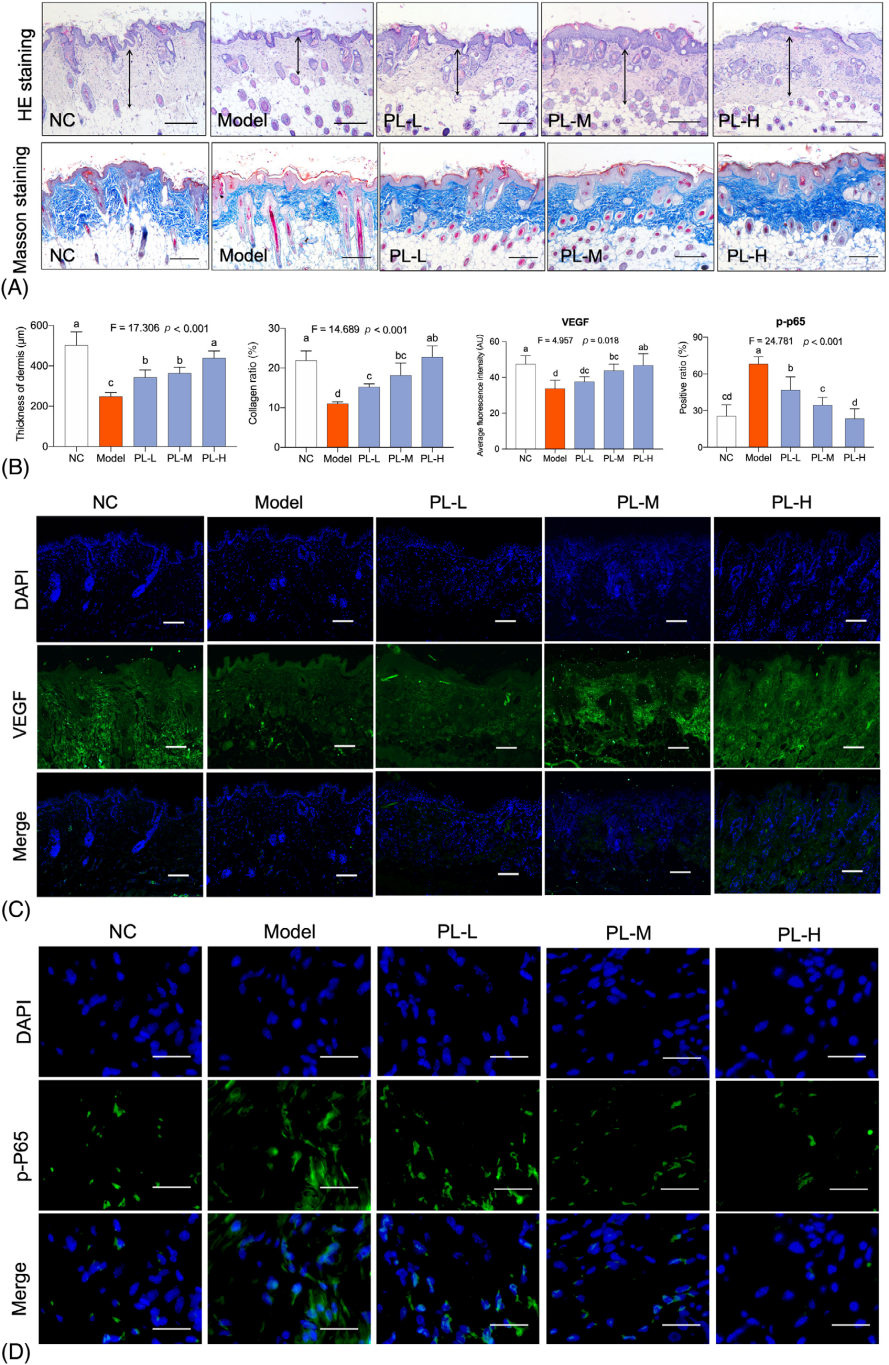
Histopathological observation using haematoxylin and eosin (HE) staining and Masson staining of mice (scale bar = 200 μm) (A). Thickness of dermis, collagen ratio average fluorescence intensity score of VEGF and positive rate of p‐P65 (B). Immunofluorescence analysis of VEGF (C) and p‐P65 (D) of mice (scale bar = 200 μm for VEGF, scale bar = 25 μm for p‐P65). Data expressed as mean ± SD. Different symbols (a, ab, b, bc, c, cd and d) indicate significant difference among groups (Fisher's least significant difference, *p* < 0.05), and the values decrease with the order from a to d. We repeated the experiments three times to ensure the accuracy of the experiments

Histopathological observation was performed on dermal thickness and collagen fibres of nude mice by HE and Masson's trichrome staining. As shown in Figure 3A,B, the dermal thickness levels of model group were significantly decreased as compared with that of NC group (*p* < 0.01 vs. NC), while that of hPL groups were significantly restored to normal levels (*p* < 0.01 vs. model). Masson's trichrome staining was used to visualize the thickness of collagen fibres. As shown in Figure 3A,B, the collagen fibres were obviously disrupted with significant decrease of thickness levels, when compared with that of NC group (*p* < 0.01 vs. NC). In contrast, the abnormalities were remarkably restored with significant increase of collagen thickness levels in hPL‐treated groups (*p* < 0.01 vs. model). Immunofluorescence result of VEGF was shown in Figure 3B,C. The skin VEGF expression was significantly decreased in model group, when compared with that of NC group (*p* < 0.01 vs. NC). In contrast, The VEGF expression of hPL groups was significantly increased and approached to the normal level, when compared with that of model group (*p* < 0.05 or *p* < 0.01 vs. model). Immunofluorescence result of p‐P65 on skin tissue was shown in Figure 3B,D. The skin p‐P65 expression was significantly elevated in model group, when compared with that of NC group (*p* < 0.01 vs. NC). In contrast, the p‐P65 expression of hPL groups was significantly lowered down towards NC level, when compared with that of model group (*p* < 0.01 vs. model). The above effects of hPL on dermal thickness, collagen production, VEGF expression and p‐P65 expression of skin were all dose‐dependent, and the p‐P65 result indicated that NF‐κB pathway might mediate the mechanism of hPL.

### 3.4 In vitro effects of hPL on senescent HDFs

**FIGURE 4:**
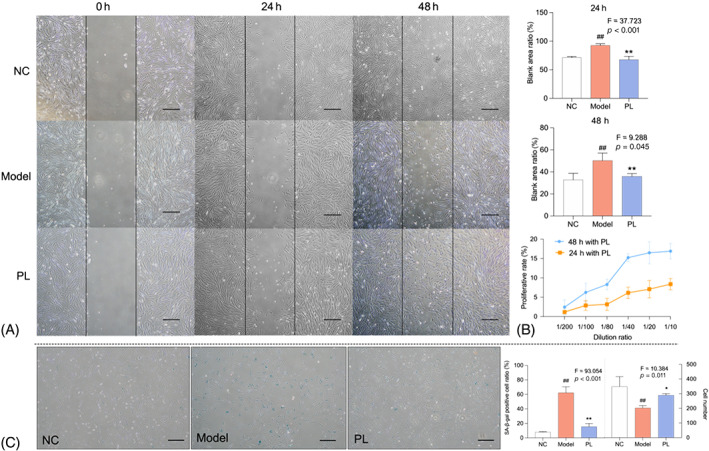
Wounding healing assay of human dermal fibroblasts (HDFs) with human platelet lysate (hPL) treatment at 0, 24 and 48 h (A). Cell migration is represented as the ratio of the scratched wound area of HDFs with 24 and 48 h PL treatment to the area without treatment (0 h). Cell viability of D‐gal induced HDFs at 24 and 48 h after hPL treatment (B). SA‐β‐gal staining of HDFs with hPL treatment and scoring the SA‐β‐gal positive cells number (C). Scale bar = 200 μm. Data expressed as mean ± SD. ^##^ *p* < 0.01 vs. NC group; *p < 0.05 or **p < 0.01 vs. model group by one‐way ANOVA followed by least significant difference multiple comparison. We repeated the experiments three times to ensure the accuracy of the experiments

D‐gal was applied to establish senescence model of HDFs, and CCK‐8 assay, wound healing assay and SA‐β‐gal staining were respectively performed to evaluate the pro‐migrative, proliferative, and anti‐senescent effects of hPL on HDFs. As shown in Figure 4B, hPL at a dilution rate ranging from 1/200 to 1/10 exerted significantly proliferative effect on HDFs in a dose‐dependent manner after 24 and 48 h treatment. Accordingly, hPL at a dilution rate of 1/40 was chosen as an effective dose for further use. As shown in Figure 4A, the blank ratios of wound area at 24 and 48 h to the area without treatment (0 h) of model group were significantly higher than that of NC group (both *p* < 0.01), and that of hPL group were significantly lower than that of model group (both *p* < 0.01). SA‐β‐gal staining is a way to exhibit senescent cells. As shown in Figure 4C, the number of SA‐β‐gal stained cells (in blue colour) of model group was significantly higher than that of NC group (*p* < 0.01), and that of hPL group was significantly lower than that of model group (both *p* < 0.01). The above data revealed that hPL effected on HDFs by inducing proliferation and migration, as well as suppressing senescence.

### 3.5 Molecular actions of hPL on senescent HDFs

**FIGURE 5:**
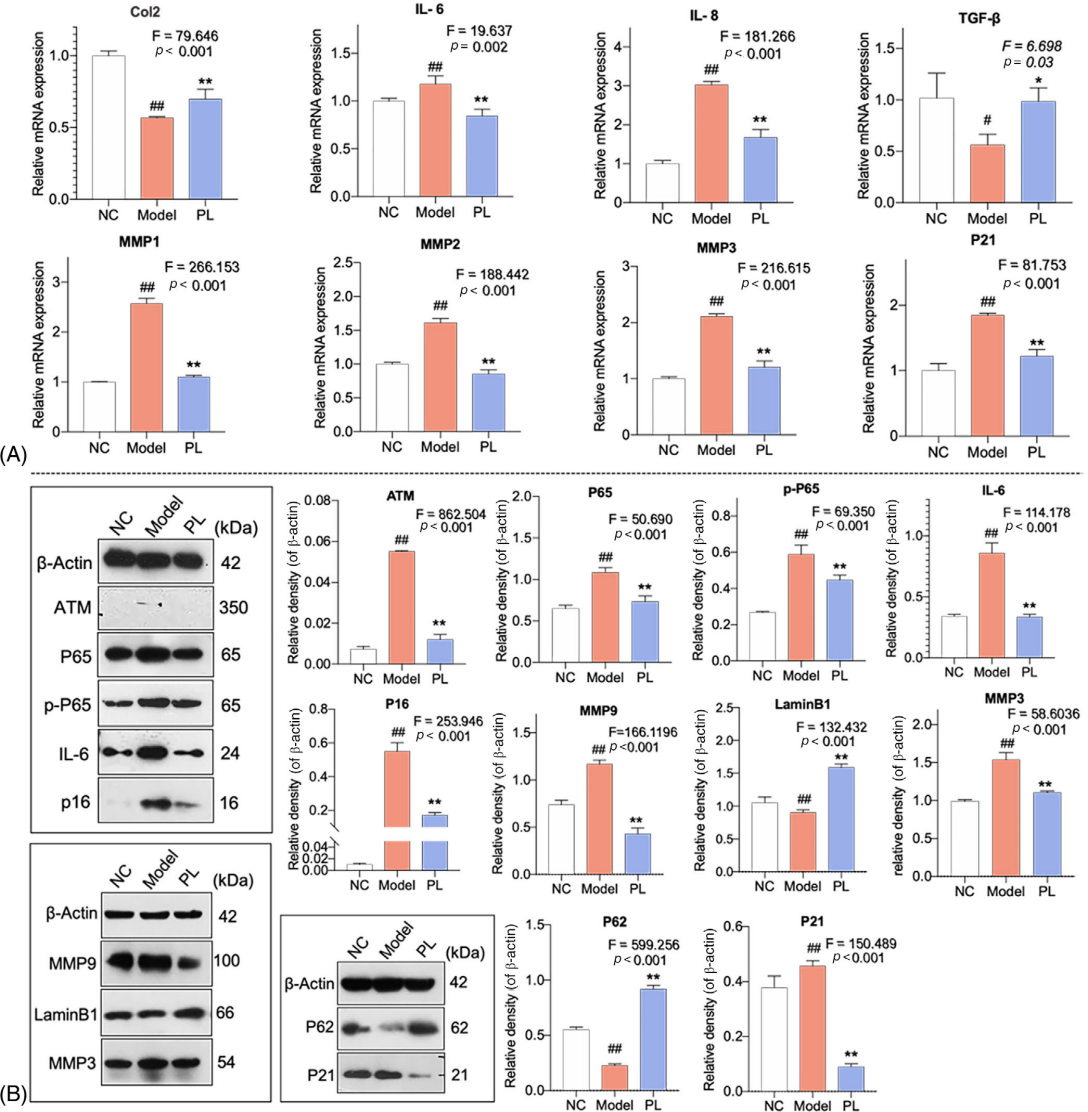
The relative mRNA expressions of genes of human dermal fibroblasts (HDFs) with human platelet lysate (hPL) treatment detected by qPCR (A). Protein bands and protein expression in HDFs with PL treatment (B). Data expressed as mean ± SD. ^##^ *p* < 0.05 or ^##^ *p* < 0.01 vs. NC group; *p < 0.05 or **p < 0.01 vs. model group by one‐way ANOVA followed by least significant difference multiple comparison. We repeated the experiments three times to ensure the accuracy of the experiments

qPCR and WB analyses were conducted to elucidate molecular actions of hPL on senescent HDFs. As shown in Figure 5A, the mRNA expressions of *IL‐6*, *IL‐8*, *MMP1*, *MMP2*, *MMP3* and *P21* were significantly up‐regulated and that of *Col2* and *TGF‐β1* were significantly down‐regulated in model group (*p* < 0.05 or 0.01 vs. NC). The abnormal changes of these genes were significantly reversed by hPL at 24 h (*p* < 0.01 vs. model). As shown in Figure 5B, the protein expressions of ATM, P65, p‐P65, IL‐6, P16, P21, MMP3 and MMP9 were significantly up‐regulated and that of P62 and LaminB1 were significantly down‐regulated in model group (all *p* < 0.01 vs. NC). In contrast, hPL significantly restored the abnormal expressions of these proteins towards normal levels (all *p* < 0.01 vs. model). Considering the above molecules as key nodes of NF‐κB signalling, it is likely that hPL exerted anti‐ageing effects on senescent HDFs through suppression of NF‐κB signalling pathway.

### 3.6 Verification of NF‐κB pathway‐dependent mechanism of hPL

**FIGURE 6:**
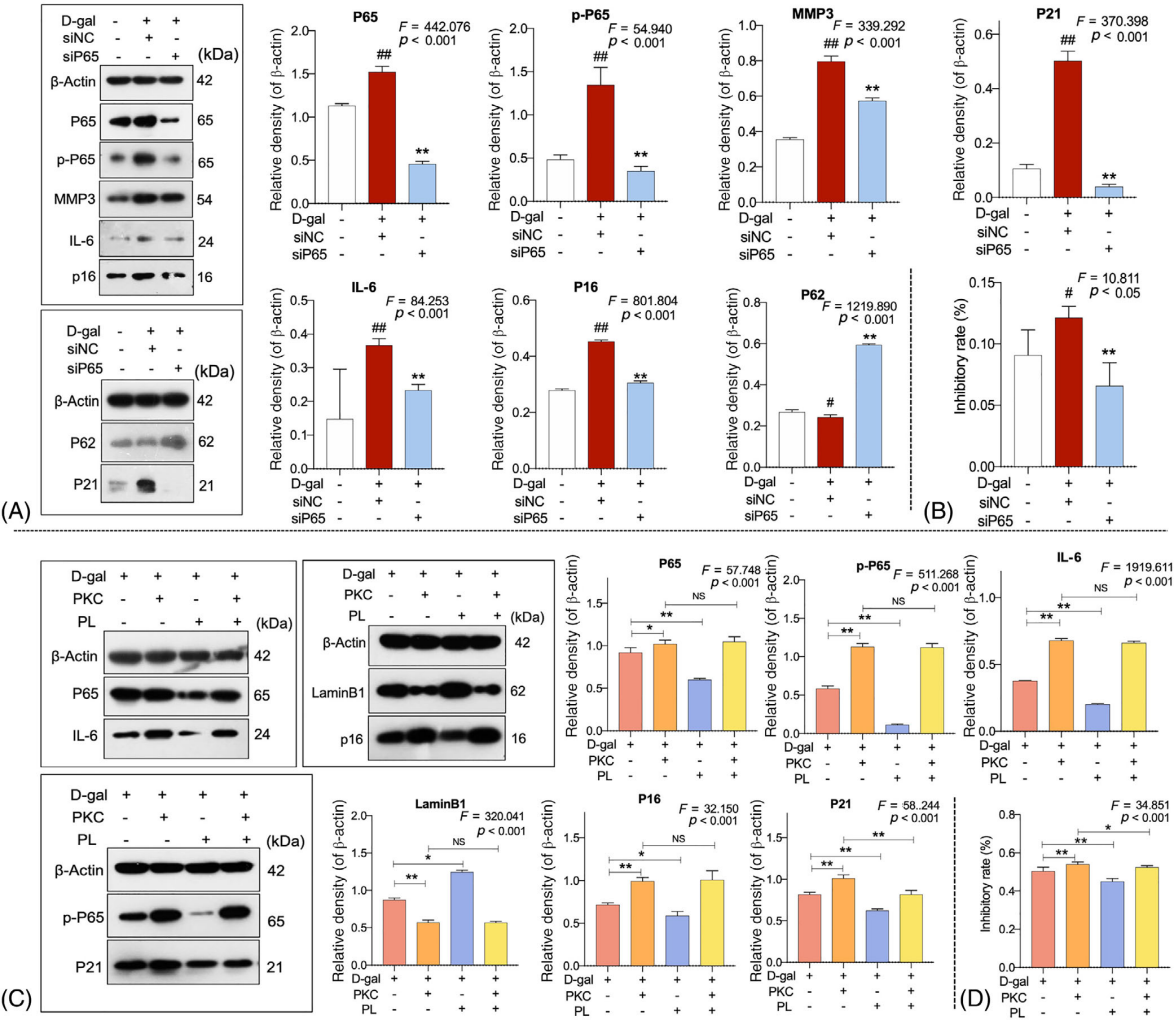
Protein bands and protein expressions of human dermal fibroblasts (HDFs) with D‐gal plus P65‐siRNA (siP65) treatment (A). Cell viability of HDFs with D‐gal and siP65 treatment at 24 h (B). siNC: non‐targeting control siRNA‐treated group; siP65: P65‐siRNA‐treated group. Protein bands and protein expressions of HDFs with human platelet lysate (hPL) and prostratin treatment (C). Cell viability of HDFs with hPL and prostratin at 24 h (D). Data expressed as mean ± SD. NS: no significant difference. *p < 0.05 or **p < 0.01 by one‐way ANOVA followed by least significant difference multiple comparison. We repeated the experiments three times to ensure the accuracy of the experiments

Positive and negative verifications were conducted to determine the NF‐κB pathway‐dependent mechanism of hPL. First, P65‐siRNA was constructed to knockdown the NF‐κB pathway of senescent HDFs, mimicking the molecular action of hPL. As shown in Figure 6A, with D‐gal plus siNC (non‐targeting control siRNA) treatment, the expression changes of senescence‐related proteins (IL‐6, P16, P21, P62 and MMP3) were similar to that of model group (all *p* < 0.05 or <0.01 vs. NC). With D‐gal plus siP65 (P65‐siRNA) treatment, the expression and phosphorylation of P65 were successfully blocked and the abnormal expressions of senescence‐related proteins were significantly restored towards normal levels (all *p* < 0.01 vs. siNC). Moreover, as shown in Figure 6B, the cell viability of HDFs was significantly inhibited by D‐gal plus siNC treatment (*p* < 0.01 vs. NC) and was significantly improved by D‐gal plus siP65 treatment (*p* < 0.01 vs. siNC). The above data indicated that siP65 exerted similar effects as hPL on molecular targets of senescence as well as cell viability of HDFs, positively verifying the NF‐κB pathway‐dependent mechanism of hPL.

As an agonist of NF‐κB, prostratin (PKC) was applied to counteract the NF‐κB pathway‐dependent action of hPL for negative verification. Four groups were designated as follows: model group with D‐gal treatment, prostratin group with D‐gal plus prostratin treatment, hPL group with D‐gal plus hPL treatment and prostratin/hPL group with D‐gal plus prostratin plus hPL treatment. As shown in Figure 6C, the expressions of NF‐κB pathway proteins (P65 and p‐P65) and senescence‐related proteins (IL‐6, P16, P21, and LaminB1) were significantly altered by prostratin, when compared with model group (all *p* < 0.01), indicating a greater extent of NF‐κB activation and senescence. Although hPL significantly restored the D‐gal‐induced alteration of senescent molecules and NF‐κB signalling pathway, the restoration on those molecules was completely counteracted by prostratin and no significant difference presented between prostratin group and prostratin/hPL group in almost all molecules (*p* > 0.05 for P65, p‐P65, IL‐6, LaminB1 and P16). Moreover, as shown in Figure 6D, cell viability data showed that the proliferative effect of hPL on HDFs was counteracted by prostratin in the prostratin/hPL group (*p* < 0.01 vs. hPL). The above results reversely verified the NF‐κB‐dependent mechanism of hPL.

### 3.7 Molecular actions of hPL‐contained growth factors on senescent HDFs

**FIGURE 7:**
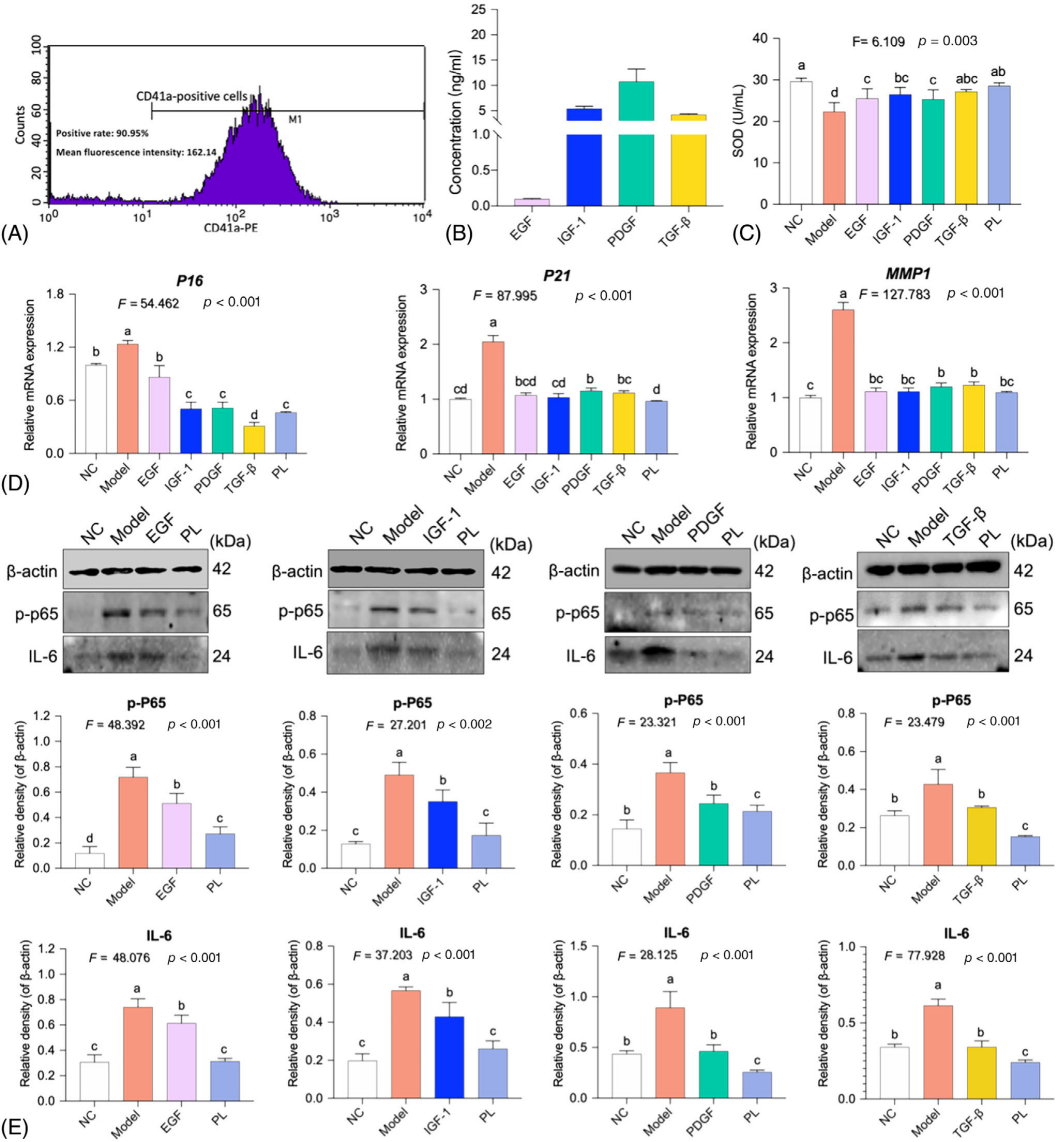
Flow cytometric pattern of CD41a expression on PRP (A) and ELISA‐tested concentration of PDGF, TGF‐β, IGF‐1 and EGF in hPL (B). Superoxide dismutase content of human dermal fibroblasts (HDFs) with treatment of human platelet lysate (hPL) or growth factors (C). Relative mRNA expressions (D) and protein expressions (E) of HDFs with treatment of hPL or growth factors. Data expressed as mean ± SD. Different symbols (a, ab, abc, b, bc, bcd, c, cd and d) indicate significant difference among groups (Fisher's least significant difference, *p* < 0.05), and the values decrease with the order from a to d. We repeated the experiments three times to ensure the accuracy of the experiments

As shown in Figure 7A, flow cytometrical analysis showed 90.95% positive expression of CD41a in hPL production before freeze–thaw lysis. As shown in Figure 7B, after lysis, hPL was obtained containing 10.46 ± 2.89 ng/ml PDGF, 4.25 ± 1.27 ng/ml TGF‐β, 5.40 ± 0.49 ng/ml IGF‐1 and 0.97 ± 0.09 ng/ml EGF. The similar concentrations of EGF (0.97 ng/ml), IGF‐1 (5.40 ng/ml), PDGF (10.46 ng/ml) and TGF‐β (4.25 ng/ml) were applied for the use of the corresponding recombinant growth factors in the following assays. As shown in Figure 7C, hPL and the four growth factors significantly up‐regulated the level of SOD (all *p* < 0.01), in which hPL achieved the highest level, followed by TGF‐β, IGF‐1, EGF and PDGF in a decreasing order, indicating a synergistic contribution of those growth factors to the anti‐oxidative effect of hPL.

qPCR and WB assays were conducted to elucidate the molecular actions of the growth factors on HDFs. As shown in Figure 7D,E, both hPL and the growth factors significantly down‐regulated the mRNA expressions of *P16*, *P21* and *MMP1* as well as the protein expressions of p‐P65 and IL‐6 (each *p* < 0.01 vs. model). The results confirmed that IGF‐1, EGF, PDGF and TGF‐β exerted hPL‐like actions on senescent molecules and NF‐κB signalling, suggesting a synergistic contribution of those growth factors to the molecular action and mechanism of hPL.

## 4 DISCUSSION

Our previous studies have reported that hPL contained PDGF, TGF‐β, IGF‐1, EGF and FGF, and possessed beneficial effects on MSCs as well as pro‐regenerative effect on osteoarthritic cartilage.^28^, ^29^ Some other reports indicated that hPL contributed to skin regeneration in wound.^30^, ^41^ Thus, hPL is regarded as a pro‐regenerative therapeutic for skin and soft tissue repair. Little study has to date investigated the rejuvenative or anti‐ageing effect of hPL. To bridge this gap, the present study first evidenced the rejuvenative efficacy of hPL against skin ageing through retrospective analysis and animal experiment, and clarified its underlying mechanism by cellular and molecular assays. The clinical and animal data demonstrated that hPL exerted pro‐regenerative and anti‐oxidative capacity on ECM matrix of aged skin, resulting in removal of wrinkles and smooth of skin texture. The cellular mechanism of hPL was mediated by inhibition of inflammation, decreased secretory of SASP and reversal of senescent state of HDFs, while the molecular mechanism was dependent on NF‐κB signalling pathway. In sum, the innovative and unique points of our study include: (1) determination of rejuvenative efficacy of hPL on skin ageing; (2) clarification of NF‐κB signalling pathway‐dependent anti‐ageing mechanism of hPL on senescent HDFs and (3) clinical verification of efficacy and safety of hPL on skin ageing.

In vivo, hPL exerted pro‐regenerative effect against skin ageing by dose‐dependently increasing dermal thickness and collagen fibres of nude mice and VEGF expression of HDFs (Figure 3). Collagen fibres are major component of dermal ECM, providing tensile strength and elasticity to keep vigorous and youthful state of skin.^42^ VEGF is a key growth factor not only for angiogenesis, but also for skin tissue regeneration, which stimulates proliferation and migration of dermal fibroblasts to producing dermal ECM.^43^, ^44^ Impairment of VEGF expression could enhance the collagen degradation and ageing of skin, while enhancement of VEGF expression exhibited anti‐ageing effect on skin.^38^, ^45^ In this study, VEGF participated in the hPL‐induced rejuvenative process of skin. Meanwhile, hPL exerted anti‐oxidative effect on aged skin by reversing SOD and MDA activities (Figure 2). An anti‐oxidative defence mechanism is crucial for preventing ROS damage and ageing stress on skin.^7^ SOD is an important anti‐oxidative enzyme forming the first defence against ROS, and MDA is a production of ROS related reactions.^46^ As a famous antioxidant, resveratrol has been reported to enhance SOD and decrease MDA, resulting in suppression of inflammatory process and improvement of collagen production of aged skin.^10^, ^47^, ^48^ In vitro, hPL exerted anti‐ageing effect on senescent HDFs by inducing cell proliferation and migration, inhibiting inflammatory state and secretion of SASP molecules, and altering senescent markers of HDFs (SA‐β‐gal, P16, P21 and LaminB1) (Figures 4 and 5). Secretion of SASP is a key feature of cellular senescence, including IL‐6, IL‐8, MMPs (MMP‐1/2/3/9), and so on to senesce neighbouring normal cells and deteriorate ECM environment.^49^, ^50^ The SASP cytokines IL‐6 and IL‐8 are hallmarks of inflammation that reinforce the senescent growth arrest and induce inflame‐ageing phenotype of nearby cells.^51^, ^52^ As a superfamily of matrix metalloproteinases, MMPs act as another kind of SASP factor to degrade ECM proteins and accelerate skin ageing.^53^, ^54^ For example, MMP‐1 acts as a collagenase, MMP‐3 acts as a stromelysin and MMP‐2 and MMP‐9 act as gelatinases, all of which contribute to the degradation of fibrillar collagen, collagen type I/II and elastin fibrils of skin.^55^, ^56^, ^57^ Inhibition of SASP is effective in promoting skin rejuvenation against ageing, resulting in decreased senescence of HDFs and enhanced synthesis of ECM proteins.^58^ Besides the SASP, increased β‐galactosidase activity is also a typical phenotype of senescence, which can directly be observed through SA‐β‐gal staining.^59^ Up‐regulations of P21 and P16 are genotypic characteristics responsible for the initiation of cellular senescence, leading to durable cell growth and cell cycle arrest.^60^ Moreover, LaminB1 acts as a major structural component of nucleus, reduction of which alters chromatin reconfiguration and thereby triggers senescence.^61^

Cellular senescence is induced by diverse stimuli, and activation of DNA damage response (DDR) pathways is involved in both initiation and maintenance of the senescence.^62^ NF‐κB signalling pathway is such a pathway modulating SASP molecules (IL‐6, IL‐8 and MMPs) in senescent cells.^63^, ^64^ It has been reported that activation of NF‐κB in young HDFs mimicked a replicative senescence of HDFs and resulted in degradation of collagen type I, suggesting an important role of NF‐κB pathway in skin ageing.^63^ In this study, the protein expressions of ATM, P65 and phosphorylated P65 were up‐regulated in senescent HDFs and reversed by hPL treatment, indicating NF‐κB pathway as the mediator of hPL's anti‐senescent mechanism on skin (Figure 5). ATM is a DDR kinase acting as an initial driver of NF‐κB pathway. It reduces P62 expression and then activates the transcription factor NF‐κB to facilitate senescence and initiate SASP.^64^, ^65^, ^66^ P62 participates in the degradation of autophagosomes in response to stimuli and negatively activates NF‐κB (subunit P65).^67^, ^68^ P65 is a major transcription factor that accumulates on chromatin of senescent cells and drives overexpressions of SASP molecules.^69^ SASP is the main characteristics of cellular senescence, containing specific pro‐inflammatory and senescence messaging secretome.^70^ The NF‐κB‐activated SASP factors, such as IL‐6, IL‐8 and MMPs, induce paracrine DNA damage, chronic inflammation and senescence of healthy neighbouring cells, resulting in acceleration of ageing.^71^, ^72^, ^73^ Therapeutic inhibition of overexpressed SASP factors (IL‐6, IL‐8, MMP‐1 and MMP‐9) of senescent HDFs could reverse inflammatory response and promote skin rejuvenation.^74^, ^75^, ^76^ By using P65 blocker (P65 siRNA) and P65 agonist (prostratin), this study confirmed the NF‐κB pathway‐dependent anti‐ageing mechanism of hPL. Moreover, the cellular experiments of this study employed a D‐gal model at 24 h in accordance with the previous reports.^38^, ^77^, ^78^ Only 24 h modelling might result into an acute toxicity in cells by oxidative stress and thereby influenced the result. To confirm the reliability of our data, we further conducted experiments by using another D‐gal model with 48 h modelling time and found similar tendency with our data at 24 h (Figure S1).

Interestingly, this study discovered that hPL‐contained IGF‐1, TGF‐β, PDGF and EGF synergistically contributed to the anti‐ageing efficacy and mechanism of hPL. Each of these growth factors at the corresponding concentration in hPL exerted hPL‐like anti‐ageing efficacy and molecular actions via NF‐κB signalling pathway, and none of them could achieve or surpass the efficacy of hPL (Figure 7). It is reasonable because these growth factors all have potential of inducing skin rejuvenation, and their combinations of them may synergistically result in the best outcome. For instance, IGF‐I is capable of maintaining skin surface lipids and thickness,^79^ and EGF, PDGF and TGF‐β are capable of proliferating HDFs, producing collagens and inhibiting MMPs of ECM, benefiting wrinkle removal and skin smooth.^80^, ^81^, ^82^, ^83^ Moreover, PDGF is a major component of adipose derived stem cells (ADSCs) secretome, which endows ADSCs anti‐ageing function on skin.^84^ To date, the concrete roles and contributions of these grown factors in hPL or PRP remain to be elucidated. Whether some other growth factors or ingredients in hPL also contribute to hPL, warrants further investigations. As the next generation of PRP, hPL possesses better bioactivity and bioavailability than PRP, since hPL contains higher concentrations of growth factors.^28^ Nevertheless, the comparison between hPL and PRP in anti‐ageing or regenerative applications has never been reported. Hypodermic injection of PRP has been reported to promote the production of elastin and fibrillin of skin.^85^, ^86^ However, PRP may also cause skin erythema, inflammation and fibrosis, which indirectly accelerates ageing process in some cases and sometimes exerts ineffective effect on skin ageing.^86^, ^87^, ^88^ Such defects may be due to the remain of heterologous activators and cell residuals (WBC and RBC) in PRP. In this study, the lysis and purification processes in hPL preparation not only released high concentrations of growth factors with no need of heterologous activators, but also removed cell residuals completely, thereby overcoming the defects of PRP. The clinical data also verified the safety of hPL in use on subjects. Moreover, hPL exerted anti‐ageing effects on skin from various aspects including dermal ECM regeneration and anti‐oxidization, wrinkle removal and texture smooth, and HDFs protection from inflammation, and senescence, whereas PRP can only ameliorate wrinkles and texture of skin in clinic with little report on anti‐senescent and anti‐oxidative effects on skin cells.^26^, ^86^ Therefore, hPL is an ideal therapeutic for anti‐ageing application on skin.

## 5 CONCLUSION

This study demonstrated hPL's efficacy on skin ageing of nude mice and clarified its mechanism relying on NF‐κB signalling pathway of HDFs. A retrospective study further verified hPL's efficacy and safety in clinic. hPL exerted beneficial effects not only on skin ECM by removing wrinkles and smoothing skin texture, but also on HDFs by suppressing oxidation, inflammation, SASP secretion and senescent state of HDFs. Although hPL is derived from PRP, it possesses better bioactivity and bioavailability and overcomes the defects of PRP, due to the high concentration of growth factors released from hPL. Nevertheless, the concrete roles and contributions of each grown factor in hPL remain unclear, warranting further investigations. Altogether, this study first revealed novel knowledge of hPL's anti‐ageing efficacy and mechanism on skin, providing an ideal therapeutic for skin ageing treatment.

## CONFLICT OF INTEREST

The authors have declared no conflict of interest.

## AUTHOR CONTRIBUTIONS

Ting Li conducted the main work of this study; Haishan Lu contributed to the clinical retrospective analysis of hPL and funding support of this study; Li Zhou contributed to the mechanism study of hPL; Ming Jia contributed to the funding support of this study; Lei Zhang improved the experimental design and methodology; Huiling Wu provided ideas and designed this study; Letian Shan designed, drafted, and funded this study; Thomas Efferth improved the design and writing of this study.

## Supporting information


**Figure S1** The relative mRNA expressions of genes of HDFs modelled by D‐gal for 48 h and treated with hPL (A). Protein bands and protein expression in HDFs modelled by D‐gal for 48 h and treated with hPL (B). Data expressed as mean ± SD. ^**##**^ *p* < 0.05 or ^**##**^ *p* < 0.01 vs. NC group; *p < 0.05 or **p < 0.01 vs. model group by one‐way ANOVA followed by LSD multiple comparison. We repeated the experiments three times to ensure the accuracy of the experiments.

## Data Availability

The data that support the findings of this study are available from the corresponding author upon reasonable request.
