# Supplementary figures and images for: Growth factors‐based platelet lysate rejuvenates skin against ageing through NF‐κB signalling pathway: In vitro and in vivo mechanistic and clinical studies
Source: Cell Prolif. 2022 Mar 11;55(4):e13212. doi: 10.1111/cpr.13212 (PMC9055903; doi:10.1111/cpr.13212)

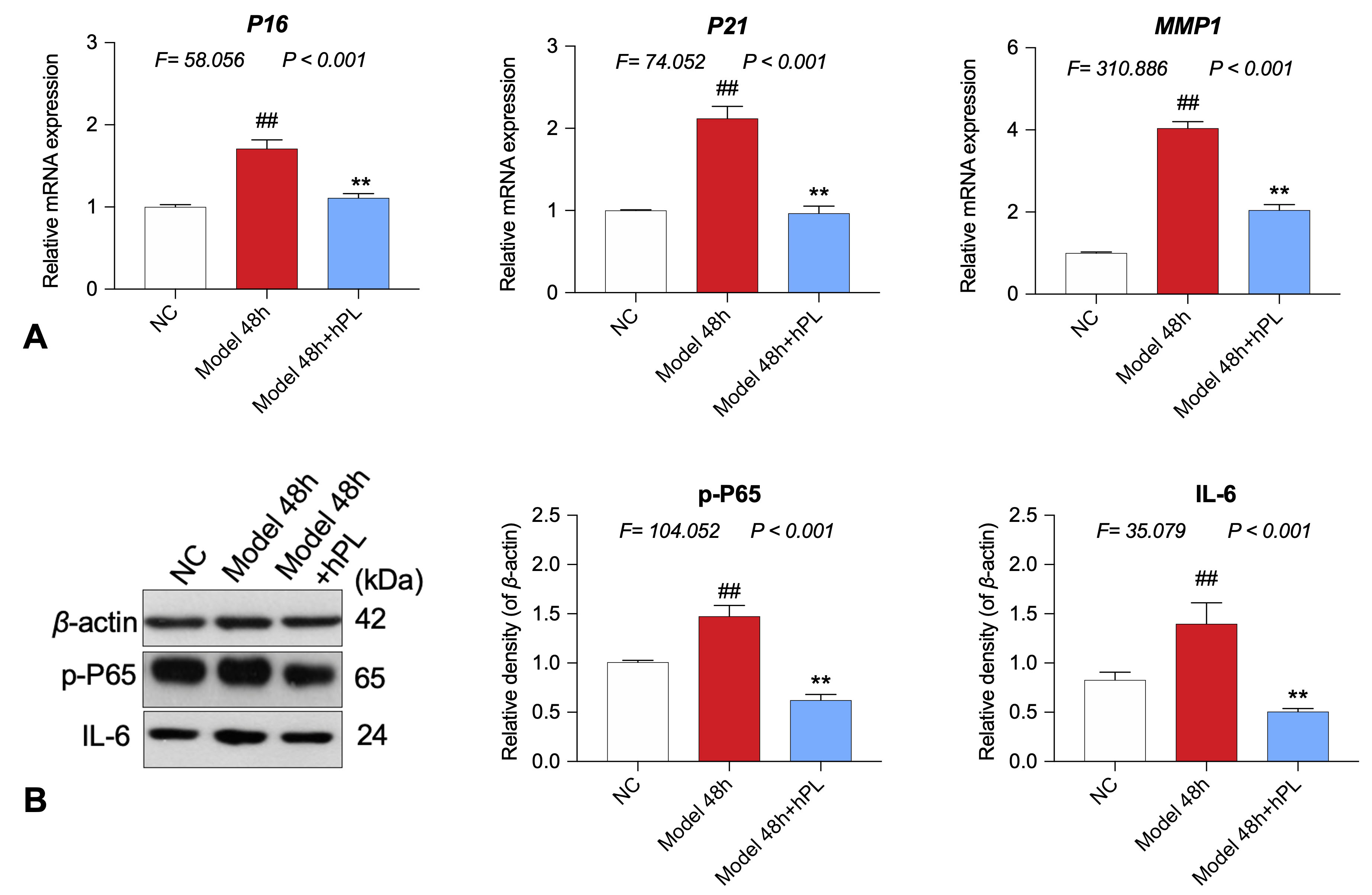

Supplement: Supplementary file 1 — Figure S1 The relative mRNA expressions of genes of HDFs modelled by D‐gal for 48 h and treated with hPL (A). Protein bands and protein expression in HDFs modelled by D‐gal for 48 h and treated with hPL (B). Data expressed as mean ± SD. ## p < 0.05 or ## p < 0.01 vs. NC group; *p < 0.05 or **p < 0.01 vs. model group by one‐way ANOVA followed by LSD multiple comparison. We repeated the experiments three times to ensure the accuracy of the experiments. [file CPR-55-e13212-s001.jpg]
